# Automated segmentation of colorectal liver metastasis and liver ablation on contrast-enhanced CT images

**DOI:** 10.3389/fonc.2022.886517

**Published:** 2022-08-11

**Authors:** Brian M. Anderson, Bastien Rigaud, Yuan-Mao Lin, A. Kyle Jones, HynSeon Christine Kang, Bruno C. Odisio, Kristy K. Brock

**Affiliations:** ^1^ Department of Imaging Physics, The University of Texas MD Anderson Cancer Center, Houston, TX, United States; ^2^ UTHealth Graduate School of Biomedical Sciences, The University of Texas MD Anderson Cancer Center, Houston, TX, United States; ^3^ Department of Interventional Radiology, The University of Texas MD Anderson Cancer Center, Houston, TX, United States; ^4^ Department of Abdominal Imaging, The University of Texas MD Anderson Cancer Center, Houston, TX, United States

**Keywords:** deep-learning, liver cancer, percutaneous ablation, computed tomography, biomechanical modeling

## Abstract

**Objectives:**

Colorectal cancer (CRC), the third most common cancer in the USA, is a leading cause of cancer-related death worldwide. Up to 60% of patients develop liver metastasis (CRLM). Treatments like radiation and ablation therapies require disease segmentation for planning and therapy delivery. For ablation, ablation-zone segmentation is required to evaluate disease coverage. We hypothesize that fully convolutional (FC) neural networks, trained using novel methods, will provide rapid and accurate identification and segmentation of CRLM and ablation zones.

**Methods:**

Four FC model styles were investigated: Standard 3D-UNet, Residual 3D-UNet, Dense 3D-UNet, and Hybrid-WNet. Models were trained on 92 patients from the liver tumor segmentation (LiTS) challenge. For the evaluation, we acquired 15 patients from the 3D-IRCADb database, 18 patients from our institution (CRLM = 24, ablation-zone = 19), and those submitted to the LiTS challenge (*n* = 70). Qualitative evaluations of our institutional data were performed by two board-certified radiologists (interventional and diagnostic) and a radiology-trained physician fellow, using a Likert scale of 1–5.

**Results:**

The most accurate model was the Hybrid-WNet. On a patient-by-patient basis in the 3D-IRCADb dataset, the median (min–max) Dice similarity coefficient (DSC) was 0.73 (0.41–0.88), the median surface distance was 1.75 mm (0.57–7.63 mm), and the number of false positives was 1 (0–4). In the LiTS challenge (*n* = 70), the global DSC was 0.810. The model sensitivity was 98% (47/48) for sites ≥15 mm in diameter. Qualitatively, 100% (24/24; minority vote) of the CRLM and 84% (16/19; majority vote) of the ablation zones had Likert scores ≥4.

**Conclusion:**

The Hybrid-WNet model provided fast (<30 s) and accurate segmentations of CRLM and ablation zones on contrast-enhanced CT scans, with positive physician reviews.

## Introduction

### Colorectal cancer in the United States

Colorectal cancer (CRC) is the third most common cancer in the United States in both men and women (1) and a leading cause of cancer-related death worldwide (2). The main cause of death for CRC patients is metastasis (3). Up to 60% of patients develop colorectal liver metastasis (CRLM) over the course of their disease, with 25% presenting with CRLM at diagnosis (4). Such facts highlight the importance of liver-directed loco-regional therapies (LRT) for these patients.

While several treatment options are available for CRLM (particularly radiation and ablation therapies), they all rely on accurate estimation of disease extent, usually involving cross-sectional imaging with contrast-enhanced CT (CECT) or MRI. CRLM often appears as hypo-enhancing lesions on routine CECT portal-venous phase images. However, their detection can be challenging owing to ill-defined margins, particularly for sub-centimeter lesions.

Both radiofrequency and microwave ablation interventions aim for a minimum margin to be achieved around the disease to ensure that all microscopic disease is treated. This requires both segmentation of disease on pre-treatment images and the ablation zone on post-treatment images to assess the ablation margin (5). The ablation zone is hypo-enhanced on CECT images, similar to the CRLM. A clinical trial is underway (Identifier: NCT04083378) to map the CRLM from pre-treatment to post-treatment imaging and assess treatment efficacy, but manual segmentations of both the disease and the ablation zone are still required (6), adding time to the procedure.

To date, automated liver disease segmentation tasks either have largely focused on primary liver disease, or have not included qualitative evaluation of generated contours (7–10). Furthermore, hepatocellular carcinomas tend to have enhancement during the arterial phase of contrast-enhanced CT with a hypodense rim (11, 12), while CRLM often shows hyperenhancement on the rim and a hypo-enhancing center (13, 14). New institution- and society-sponsored competitions, such as the liver tumor segmentation (LiTS) challenge (15) and the 3D-IRCADb01 (7) dataset, have included data from both primary and secondary liver cancers, enabling investigation, development, and comparison of automatic segmentation algorithms using public data.

It is hypothesized that fully convolutional neural networks, trained using novel methods to account for the challenges of varying disease size, will provide rapid and accurate identification and segmentation of both CRLM and ablation cavities. We believe that this approach will facilitate the automated detection of CRLM, radiation treatment planning for CRLM, and the evaluations of margin in ablation therapy.

## Materials and methods

### Quantitative training, validation, and testing

To ensure reproducibility by other institutions, data were provided by the publicly available LiTS challenge (15). LiTS consists of CECT scans from 131 patients with primary and secondary liver disease collected from seven different institutions (15). Subjects suffered from primary tumor disease, such as HCC, as well as secondary liver tumors and metastasis from breast, lung, and CRC. Ground-truth segmentations of the liver and disease were provided in the data; the goal of the model is to similarly segment the disease. Each image was reviewed by BMA, BCO to remove the data showing hyper-enhancing metastases, or lacking image acquisition parameters. A total of 92 patients remained.

The model was evaluated *via* submission to the LiTS challenge and the 3D-IRCADb01 publicly available dataset of 20 patients (10 male and 10 female patients) with liver disease (16). **Table 1** shows the image acquisition parameters for the training, validation, and test sets. Five of the patients from the 3D-IRCADb01 dataset were excluded: patients 5, 11, and 20 had no disease; patient 12 had a large cystic lesion at the base of the liver; and patient 18 had CRLM with atypical enhancement pattern.

**Table 1:** Image acquisition parameters of the LiTS challenge for the training, validation, and test sets.

Origin	Distribution	Mean (min–max)		
		Slice thickness (mm)	Pixel size X (mm/voxel)	Pixel size Y (mm/voxel)
LiTS	Training (*n* = 72)	1.64 (0.7–5.0)	0.75 (0.60–0.98)	0.75 (0.60–0.98)
	Validation (*n* = 20)	2.02 (0.7–5.0)	0.78 (0.68–0.98)	0.78 (0.68–0.98)
	Test (*n* = 70)	2.43 (0.45–6.0)	0.75 (0.60–0.98)	0.75 (0.60–0.98)
3D-IRCADb	Test (*n* = 15)	1.78 (1.0–4.0)	0.72 (0.56–0.87)	0.72 (0.56–0.87)

### Data pre-processing

#### Image intensity manipulation

A patient-specific mean and standard deviation Hounsfield unit was calculated for normalization on the basis of the full width at half maximum of the values within the liver; this reduced outliers as compared to using a global mean and standard deviation. The image intensity outside of the masked liver was set to be equal to 0.

#### Voxel size resampling

All training and validation images and ground-truth segmentation were resampled to 1 mm slice thickness, and 0.75 mm in the axial plane, using bi-linear interpolation.

#### Training image “slabs”

Initial training on the entire patient liver resulted in a model that struggled to identify disease sites. We believe that this is due to the disparity in class representation, being that a majority of the liver is “normal” and the model could achieve a high segmentation accuracy by segmenting everything as “normal”. Simple class weighting would not solve this problem as it would result in the model weighing cases with extensive disease cases as more important than the less extensive disease cases.

**Figure 1:**
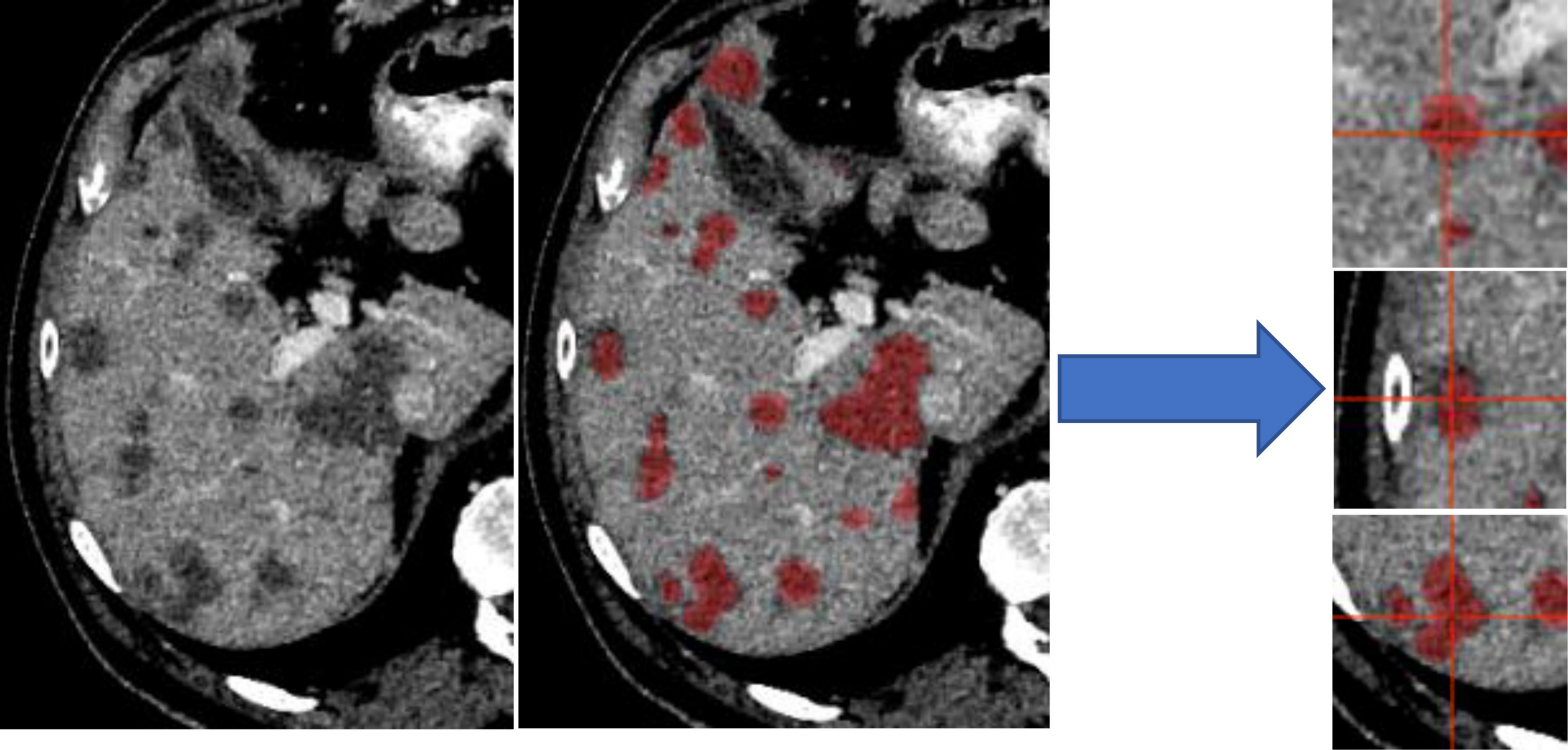
Liver distributed into individual slabs of 32 × 120 × 120. Disease was labeled as disease, regardless of the center of the slab. The validation and test set were not broken into slabs; our architectures accepted variable input sizes, with the entire liver being passed at once for evaluation and testing.

To account for disparities in class representation, where smaller structures (CRLM) are inherently “worth less” than large structures (normal liver), training was distributed into unique “slabs”. Each independent disease site was divided into “slabs” of 32 × 120 × 120 voxels. This size was selected arbitrarily as a balance of encompassing a large section of liver while reducing memory requirements. This ensures a representation of both disease and normal liver in each training step. **Figure 1** illustrates several disease slabs for one patient. After extraction, the training dataset consisted of 572 unique samples.

The validation and test set were not broken into slabs, with the entire liver being passed at once for evaluation and testing.

### Architectures

**Figure 2:**
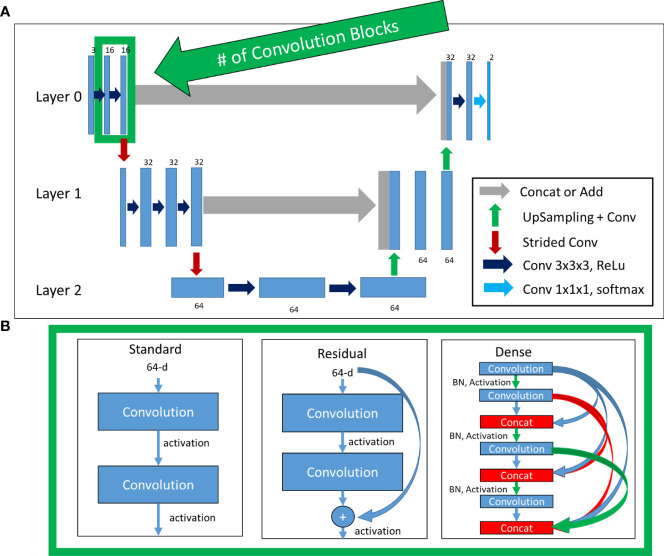
Top **(A)**: Basic architecture framework. Bottom **(B)**: Difference in convolution blocks, surrounded by green to indicate the same region in **(A)**. Standard: previous feature maps are convolved and activated. Residual: previous feature maps are directly added to convolutional output in a skip-connection before activation. Dense: previous feature maps are continually concatenated together before activation and convolution. BN, batch normalization.

**Figure 3:**
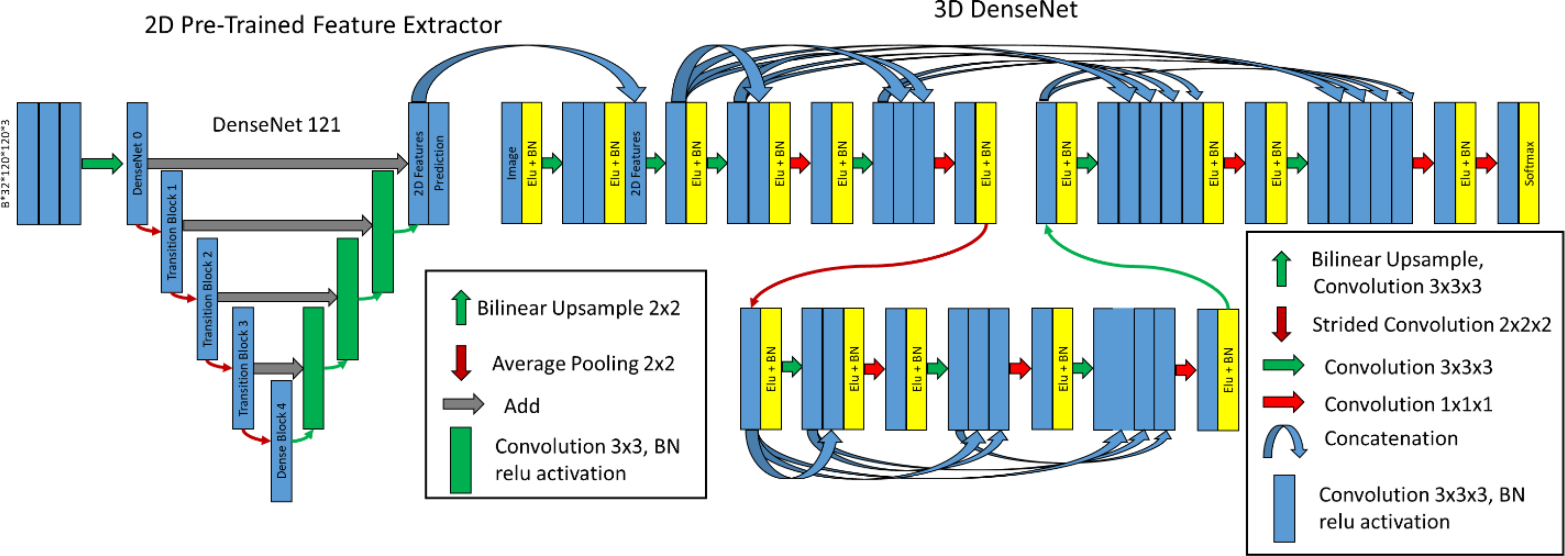
Hybrid-WNet. (Left) Pre-trained 2D DenseNet 121 converted into UNet, where final features, filters from 2D prediction, are 32 and concatenated into 3D DenseNet. (Right) 3D DenseNet architecture was defined as two layers, two convolution blocks in layer 0, three convolution blocks in layer 1, and eight initial filters. BN, batch normalization.

Four architectures were investigated: Standard 3D-UNet, Residual 3D-UNet, Dense 3D-UNet, and Hybrid-WNet (pre-trained Standard 2D-UNet with a 3D-DenseUNet). The basic framework remained the same for the Standard, Residual, and Dense 3D-UNets (**Figure 2A**). The differences in the Standard, Residual, and Dense 3D-UNets are represented in **Figure 2B**. The Hybrid-WNet architecture is shown in **Figure 3**. A list of parameters for each architecture is listed in **Table 2**.

**Table 2:** Investigated architectural hyper-parameters for each architectural style.

Parameters		Architecture style		
		Standard	Residual	Dense
Layers		1, 2, 3, 4	1, 2, 3, 4	1, 2, 3
Convolution blocks	Initial	1, 2, 3	1, 2, 3, 4	2, 3
	Increase rate	0, 1, 2	0, 1, 2	0, 1
	Maximum	4	4	4
Filters	Initial	8, 16, 32	32	8, 12, 16, 32
	Growth Rate	–	–	0, 4
	Maximum	32, 64, 128	128	128, inf

Note that the number of filters doubled after each pooling. “Growth Rate” is unique to the Dense network.

#### Residual 3D-UNet

The Standard 3D-UNet was expanded to include residual connections for each convolution block in a layer, with motivation from the “ResNet” architecture (17). Residual connections have the benefit of allowing a “flow” of loss from previous convolutions. This allows the model to create skip connections over convolutions that might not be necessary.

#### Dense 3D-UNet

A more complete “flow” of loss from previous convolutions can be realized with the DenseNet architecture (18). This architectural style allows previous convolutions to be re-used. The reuse of previous convolutions allows the number of filters to be significantly reduced; the increase in total number of filters is referred to here as the “growth rate”.

#### Hybrid-WNet

The architecture combines 2D features extracted from the pre-trained 2D DenseNet-121 (18) in Tensorflow (19) with a 3D convolutional neural network. The term Hybrid-WNet was coined on the basis of the W-shaped appearance of two UNets beside each other (**Figure 3**).

The Hybrid-WNet architectural style was inspired by Li et al. (9) with substantial alterations. First, in architecture training, the training process was broken into four steps: (1) training only the new decoding side of the DenseNet 121, (2) training the entire DenseNet121, (3) training only the 3D network with the extracted 2D features, and (4) entire end-to-end training. By breaking up the training process in this fashion, we ensured that high learning rates could be used without the risk of “untraining” pretrained layers, as was noticed by a marked dip in performance in the first iterations of subsequent training if previous layers were not frozen. Second, the 3D DenseNet contained truly dense layers, with extracted features shared throughout the entirety of each layer; this enabled the use of significantly fewer features.

### Model training

All model training was performed using NVIDIA-Tesla V100 32GB GPUs (20). All model creation, training, optimization, and evaluation was performed using Tensorflow2.2.0 (21). Models were optimized using a sparse categorical cross entropy loss (https://www.tensorflow.org/api_docs/python/tf/keras/losses/SparseCategoricalCrossentropy) and Adam optimizer. Mixed precision was enabled to reduce the training time.

The model was trained with two inputs: CT image and binary mask of the liver. The mask automatically assigns a background to any voxel outside the liver. Training involved passing B*N*H*W*C tensors to the model, where the (B)atch varied from 8 to 16, the (N)umber of slices was 32, the (H)eight was 120, the (W)idth was 120, and the (C)hannels were 2 (image and liver mask). Thus, a single pass might be 8 × 32 × 120 × 120 × 2 in size.

#### Training the DenseNet121 UNet

When training the 2D aspect of the Hybrid-WNet, 3D slabs were reshaped into stacked 2D images. For example, a batch of 8 × 32 × 120 × 120 × 1 would be transformed into 8 × 32 × 120 × 120 × 1. In the first training iteration, all weights on the pre-trained encoding architecture were frozen. Next, all weights were made trainable, allowing the model to tweak any pre-trained layers.

#### Training the combined 2D-3D WNet

After training the 2D part of the W-Net, 2D features are concatenated to the input of the 3D model. Features extracted from the 2D network would have dimensions of 8 × 32 × 120 × 120 × 32, “2D Features”, **Figure 3**. All weights from the 2D network were initially frozen, and only the 3D model trained. Next, all weights were unfrozen, allowing the model to be fine-tuned.

A visual representation of the combined architectures can be seen in **Figure 3**.

#### Hyper-parameters

For training each model, a variation in the cyclical learning rate (22) was used (GitHub link: anonymized for review), with linear increase and decrease between min and max. Optimal learning rates vary based on each architecture parameter, with the minimum and maximum learning rates identified using an in-house function (Github link: anonymized for review), **Supplementary Figure 1**. Augmentations of the training were provided in the form of flipping and mirroring the input data.

#### Model optimization

Each model was run three times using randomly initialized variables to reduce the likelihood of poor initialization. Plotnine and Tensorflow’s Tensorboard (https://github.com/tensorflow/tensorboard) was used to identify trends and direct model training. The final model was selected on the basis of the Dice similarity coefficient (DSC) between the validation set and the ground truth.

**Figure 4:**
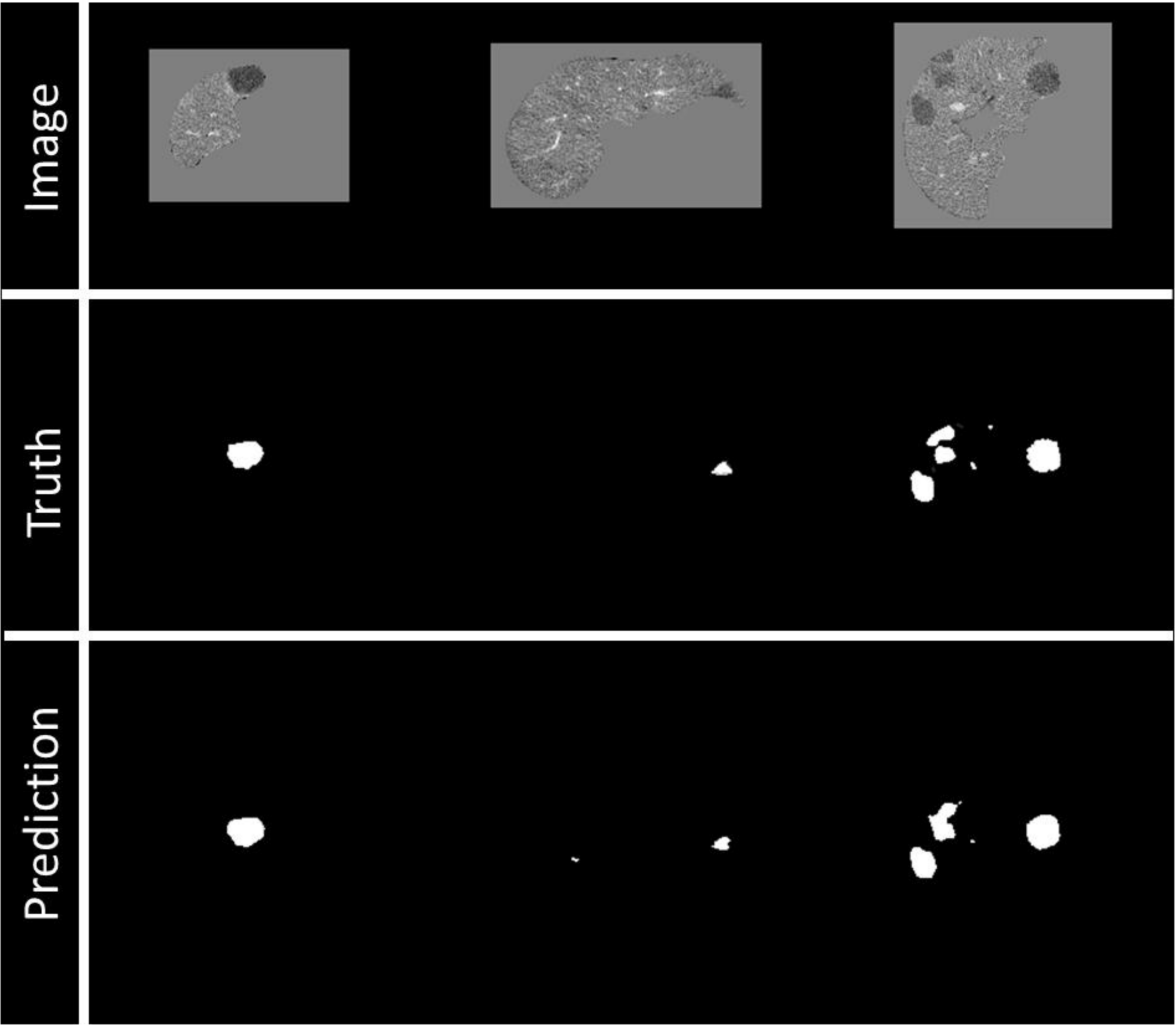
Example of visualization of prediction on validation data during training. Top row: Image being fed into the model, masked by the liver segmentation. Middle row: Ground truth of disease contours. Bottom row: Prediction of disease contours, set with a threshold of 0.5 to the binary mask.

Prediction images were visualized during training to assess the training process (**Figure 4**) as a Tensorflow callback (Github link: anonymized for review).

### Model evaluation

#### Seed and threshold values

The disease predictions from the model ranged from 0 to 1. The most inferior and superior aspects of a disease site often had a lower probability than the center of the disease. For this reason, seed-point growth was investigated for the final prediction. Seed points were created to define the likely starting point of a disease site, and then grown outwards to a threshold value. Seed values investigated from 0.25 to 0.95 in 0.01 increments, and threshold values from 0.2 to 0.8 in 0.01 increments.

#### Quantitative evaluation

Model performance was evaluated on the test set using the DSC and Median Surface Distance between the manual and predicted segmentations. Predictions were reported in a disease site-by-site, patient-by-patient, and “global” basis.

For the site-by-site comparison, each non-connected disease segmentation of the test patients was considered an independent case. Metrics were computed between the manual segmentation and the closest continuous predicted disease segmentation, using the distance between site centroids. For the patient-by-patient comparison, metrics were computed between the manual and predicted segmentations within the entire liver. For global DSC, all images were stacked together; this metric comes from the LiTS challenge (15); otherwise, DSC refers to a patient-wise evaluation. All metrics were computed using the original image resolution.

**Figure 5:**
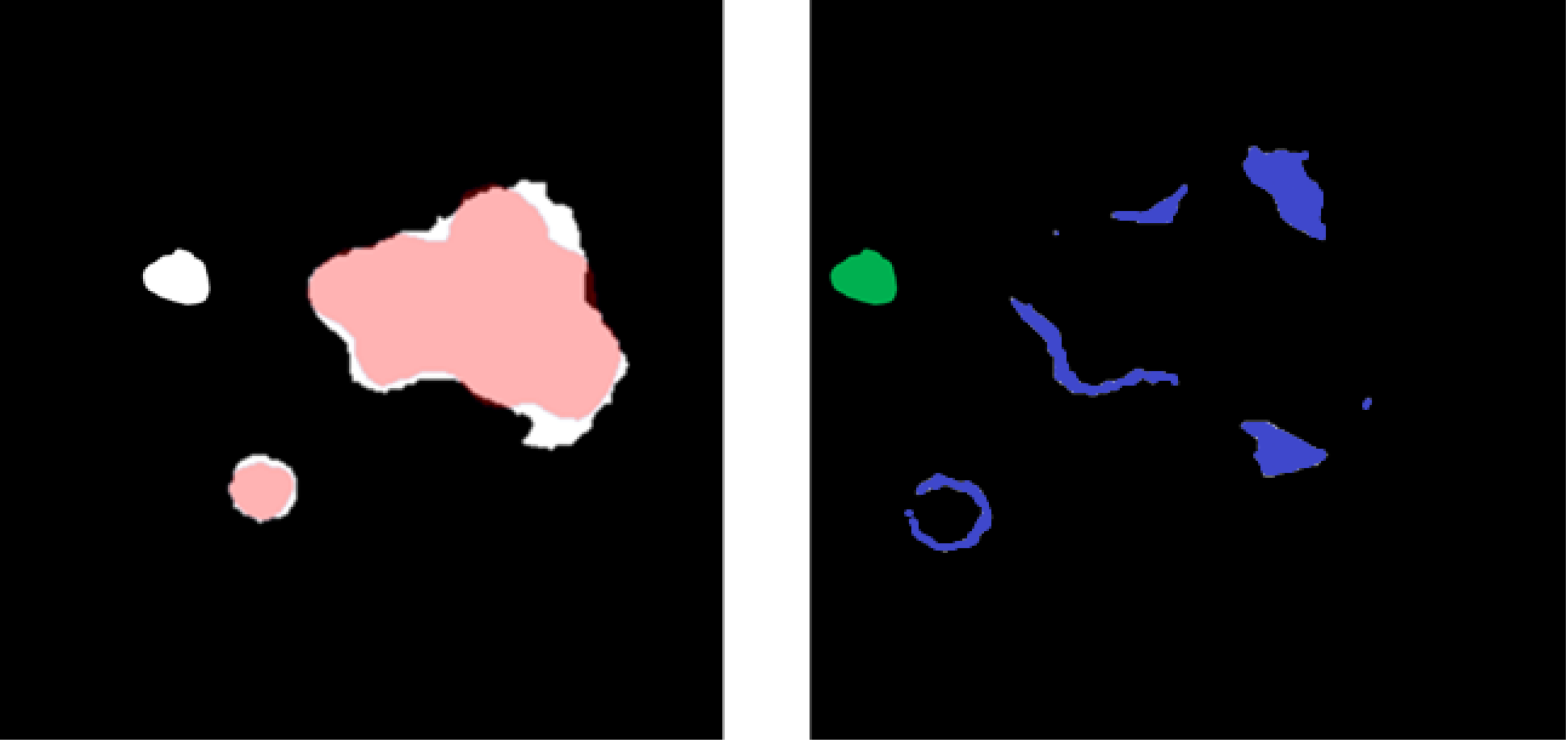
(Left) Overlay between predicted (white) and ground-truth (red) disease segmentations. (Right) Subtraction of the predicted and ground-truth disease segmentation and over-segmentation of the disease volume (blue) and unconnected erroneous false-positive region (green). The sum of blue and green is a false-positive volume.

The sensitivity of the model was evaluated on a site-by-site basis, where disease was considered identified if at least 45% of the ground truth overlapped with prediction. False-positive volume was defined as the volume of the predicted segmentation outside of the ground-truth segmentation and was composed of two errors: over-segmentation and erroneous segmentation. Erroneous false-positive volume was quantified as the volume that was completely unconnected from any ground-truth segmentations, and the over-segmentation false volume was quantified as the total predicted volume minus the erroneous volume (**Figure 5**).

#### Qualitative evaluation

For large structures, a high DSC can hide clinically important inaccuracies, while for smaller structures, a low DSC can be overly critical (23). The qualitative assessment of both CRLM and ablation segmentations was performed by a radiology-trained physician fellow (1, YML) and two board-certified radiologists, an interventional radiologist (2, BCO) and a diagnostic radiologist (3, HCK), both of whom have more than 10 years of experience. This will evaluate if the generated contours are deemed clinically useable.

Eighteen patients, who had previously undergone targeted thermal ablation therapy for colorectal liver metastasis at our institution, were retrospectively identified under an Institutional Review Board-approved study (IRB: 2019-0213); these patients had 24 CRLM sites and 19 ablation sites. The predicted CRLM and ablation contours were scored independently based on a Likert quality scale of 1–5. A breakdown of these scores can be found in **Supplementary Table 1**. A score of 4 is defined as requiring minor changes on less than four slices, or changes that would require less than 10 s to fix.

## Results

The best validation loss scores were Standard UNet: 0.041, Residual UNet: 0.024, Dense UNet: 0.016, DenseNet2D (Encoder frozen): 0.022, DenseNet2D (All trainable): 0.013, DenseNet3D (2D frozen): 0.011, and DenseNet3D (All trainable): 0.0092.

### Quantitative

The best model was the Hybrid-WNet model: the 3D-UNet contained two layers, two convolution blocks, and 32 filters and had a convolution lambda of two. The model consisted of 14,497,600 parameters (14,408,960 trainable and 88,640 non-trainable).

A seed value of 0.67 and a threshold value of 0.30 resulted in the highest overall DSC in the validation dataset. LiTS test set (*n* = 70) predictions required a mean of 9.58 s, with a standard deviation of 2.32 s.

#### Site-by-site evaluation

##### 3D-IRCADb

On a site-by-site basis, the median surface distance, DSC, and sensitivity are presented in **Table 3**. Sites are distributed into two groups based on diameter: sites < 15 mm and ≥ 15 mm. For sites ≥ 15 mm (*n* = 48), mean DSC was 0.74 and sensitivity was 98%. For sites < 15 mm (*n* = 73), mean DSC was 0.16 and sensitivity was 23%.

**Table 3:** Mean, min, and max Dice similarity coefficient and median surface distance and sensitivity for individual disease sites by differing size criteria of the 3D-IRCADb dataset.

Disease site diameter	# Sites	Dice similarity coefficient			Median surface distance (mm)			Sensitivity
		Mean	Min	Max	Median	Min	Max	
<15 mm	73	0.16	0.00	0.89	28.25	0.67	108	23% (15 of 73)
≥15 mm	48	0.74	0.00	0.94	1.23	0.28	19.4	98% (47 of 48)

##### Institutional Data

For the pre-treatment CECT CRLM target disease (*n* = 19 sites), the mean (min–max) DSC was 0.80 (0.59–0.91), with 84% (16/19) having DSC ≥ 0.76. For the post-treatment CECT target ablation zones (*n* = 14 sites), the mean (min–max) DSC was 0.75 (0.09–0.90), with 71% (10/14) having DSC ≥ 0.76.

#### Patient-by-patient evaluation

Patient-by-patient evaluation for 3D-IRCADb and institutional data are summarized in **Table 4**.

**Table 4:** Metrics of Dice similarity coefficient, median surface distance (mm), false-positive discoveries (per patient), and false-positive volume (cc) for 15 3D-IRCADb Test Patients.

Metric	3D-IRCADb Patients (*N* = 15)			
	Median	Min	Max	Standard deviation	
Dice similarity coefficient	0.74	0.40	0.89	0.16	
Global Dice similarity coefficient	0.81	N/A	N/A	N/A	
Median surface distance (mm)	1.95	0.57	8.00	1.90	
False-positive discoveries (per patient)	1	0	7	2.0	
False-positive volume (cc)	4.25	0.08	28.34	7.48	
Erroneous false-positive volume (cc)	2.35	0.00	14.56	5.01	
Over-segmentation false-positive volume (cc)	1.24	0.00	17.56	5.50	
**Metric**	**CRLM Patients (*N* = 15)**			
	**Median**	**Min**	**Max**	**Standard deviation**	
Dice similarity coefficient	0.78	0.28	0.91	0.17	
Median surface distance (mm)	0.78	0.01	83.28	27	
False-positive discoveries (per patient)	1	0	4	1.2	
False-positive volume (cc)	2.43	0.28	14.47	4.68	
Erroneous false-positive volume (cc)	1.05	0.00	14.23	4.54	
Over-segmentation false-positive volume (cc)	0.73	0.23	3.68	0.97	
**Metric**	**Ablation Patients (*N* = 9)**			
	**Median**	**Min**	**Max**	**Standard deviation**	
Dice similarity coefficient	0.79	0.42	0.89	0.16	
Median surface distance (mm)	0.76	0.01	5.72	1.76	
False-positive discoveries (per patient)	2	0	6	1.6	
False-positive volume (cc)	27.8	5.84	174.07	61.25	
Erroneous false-positive volume (cc)	5.62	0.00	107.47	32.77	
Over-segmentation false-positive volume (cc)	11.40	4.35	172.54	50.97	

##### 3D-IRCADb

Quantitative metrics on a patient-by-patient basis for 3D-IRCADb are summarized in the top of **Table 4**. Median DSC was 0.74, median surface distance was 1.95 mm, and median false-positive discoveries per patient was 1. Note that a single patient might have multiple disease sites of varying sizes. The global DSC score was 0.81.

##### Institutional data

Quantitative metrics for the institutional data are summarized in the middle and bottom of **Table 4**. For the pre-treatment CECT CRLM target disease (*n* = 15 patients), the median DSC was 0.78, median surface distance was 0.78 mm, and false-positive discoveries per patient was 1. For the post-treatment CECT target ablation zones (*n* = 9 patients), the median DSC was 0.79, median surface distance was 0.76 mm, and false-positive discoveries per patient was 2.

### Qualitative evaluation

#### Institutional data

**Figure 6:**
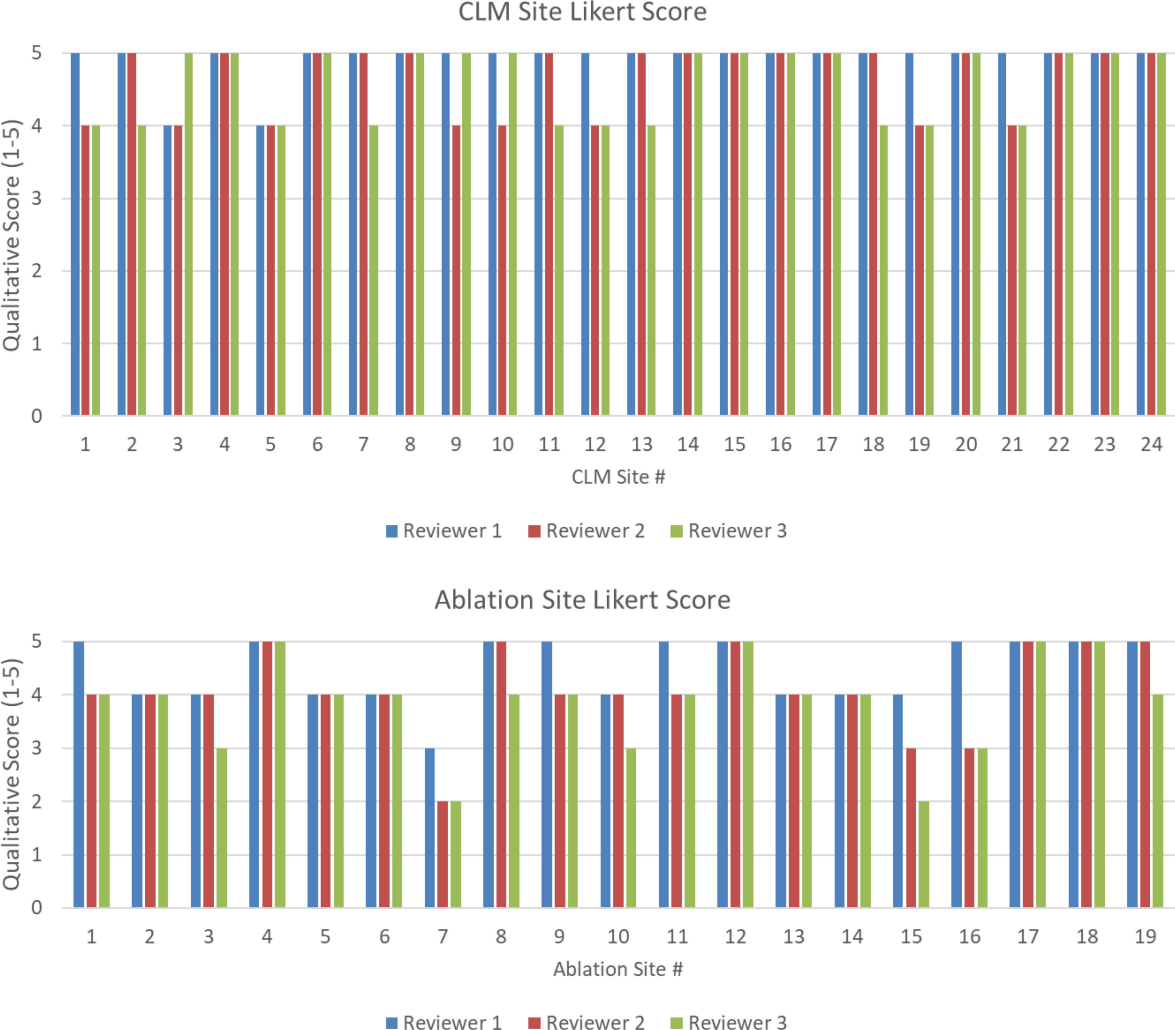
(Top) Likert score by each reviewer for each disease site. (Bottom) Likert score by each reviewer for each ablation site. A higher value is associated with higher quality.

**Supplementary Table 2** shows the Likert scores of the two radiologists and the radiology-trained physician fellow for each targeted disease and ablation zone. The majority vote mean (min–max) Likert scores for the target disease volumes (*n* = 24) were 4.8 (4–5). The majority vote mean (min–max) Likert scores for targeted ablation volume (*n* = 19) were 4.1 (2–5). All (24/24) of the CRLM contours and 84% (16/19) of the ablation contours had a majority Likert score ≥4. **Figure 6** shows the scores of the two radiologists and physician fellow on the CRLM and ablation zones.

## Discussion

While fully convolutional networks have been previously investigated for the segmentation of CRLM (8), only a quantitative assessment of model performance has been reported. A comparison of the model results to those of other authors is given in **Table 5**; the size distributions were varied to match those of previous work, and compared in both a site-by-site basis and patient-by-patient basis. While the model performed better in larger-diameter sites (≥15 mm) by DSC and sensitivity compared to Vorontsov et al. (8), it performed more poorly with sites <10 mm. Similar results have also been reported by Christ et al. (7); however, their work focused on primary liver disease.

**Table 5:** Comparison of CRLM segmentation results from our model and the literature.

Method	Model	Disease diameter and source	No. of sites	Mean DSC	Sensitivity
Presented method	Hybrid-WNet	<10 mm	42	0.00	7%
		10–20 mm	49	0.43	59%
		15–20 mm	18	0.68	94%
		≥20 mm	30	0.77	98%
		LiTS	70 patients	0.810 global	–
		3D-IRCADb	15 patients	0.69	–
Vorontsov et al. (8)	FCN	<10 mm	30	0.14	10%
		10–20 mm	35	0.53	71%
		>20 mm	40	0.68	85%
Li et al. (9)	Hybrid Dense UNet	LiTS	70 patients	0.824 global	–
Seo et al. (10)	mU-Net	3D-IRCADb	5 patients	0.68	–

Patients were specified instead of sites for LiTS test submission.

Within our institutional data, 84% (16/19) of the disease sites had a DSC > 0.76, which has been reported as the inter-observer variability for CRLM segmentation (24).

In this study, we proposed a Hybrid-WNet model architecture for the segmentation of the disease sites and ablation areas in the context of CRLM treated with ablation therapy. The model was further evaluated using the Likert scoring method by two board-certified radiologists (BCO, interventional radiologist and HCK, diagnostic radiologist) and a radiology trained physician fellow (YML), with majority voting scores ≥4 out of 5 for 100% (24/24) of the disease and 84% (16/19) of the ablation segmentations. The prediction process has been implemented in a treatment planning system (RayStation 9B, RaySearch Laboratories, Sweden) (25) and can perform segmentations in <30 s, making it suitable for clinical use.

Our work is innovative because of the Hybrid-WNet model architecture and the training of the model; the model had 98% (47/48) sensitivity on disease sites ≥15 mm, with predictions in <30 s.

The proposed Hybrid-WNet architecture in this study differs from the model that inspired it (9) by adding additions to the 2D feature extractor, and connecting all of the convolutional layers within the convolution blocks between the encoding and decoding of the 3D-DenseNet. Previously published studies proposed similar architectures that were focused only on dense connections within a single convolutional block (26–28). The proposed implementation allows the model to receive inputs from every convolution of the same image size across the entire network. This global passing of convolution layers across the network removes bottlenecks in each convolution block, something that is particularly important with dense connections where convolutions can be re-used. The proposed model will be publicly available (Github: redacted for anonymization).

Segmentation of CRLM has historically been difficult because of the relatively small size of lesions and the large search area. The extent of disease can vary from patient to patient, from a single lesion to multiple lesions. Simple class weighting of disease would lead to favoring training in patients with more disease sites. The proposed method of splitting the liver into slabs that were centered specifically on disease sites ensures that the model learns using a more balanced representation of data. The training workflow was designed to allow the model to learn from batches that contain cubic images from multiple patients at once, enabling the creation of a more generalized model.

### Limitations

Our study has several limitations. The mean sensitivity was only 7% in disease sites <10 mm in diameter compared with 98% in sites >15 mm. We believe that the low sensitivity in small sites may be partly due to the test data used, where several 10- to 15-mm disease sites were present on a single scan slice. We furthermore believe that this model should not be used as a strictly diagnostic device. If the diagnosis of smaller disease sites is wanted, the “seed” and “threshold” values, discussed in *Model evaluation*, can be reduced. Overall, this would increase sensitivity, while also increasing false positives.

Unfortunately, manual contours were not present for all the institutional CRLM and ablation data, limiting the quantitative comparison to 19 of the 24 CLRM sites and 9 of the 19 ablation sites.

Majority voting showed poor Likert score (<4) for three ablation sites. The predictions for these sites can be seen in **Supplementary Figure 2**, where disagreement about the boundary of the ablation zone and the time needed for correction resulted in a range of scores from the reviewers. We believe that suboptimal imaging quality during porto-venous CT acquisition phase might have negatively impacted ablation zone boundary identification in such patients. Optimizing imaging acquisition protocol intra-procedurally during ablation interventions might overcome this limitation.

The measurement of false-positive volume seemed to be highly biased when there were small amounts of over-segmentation on large tumors; this was the rationale for the creation of the erroneous and over-segmentation false-positive volumes. To ensure transparency, all three are shown and relied on qualitative assessment to add weight to the quality of the contours.

### Conclusions

The proposed Hybrid-WNet model provided fast (<30 s) and accurate CRLM and ablation zone segmentations for CECT. The model’s results were well accepted by the reviewers, with all three scoring the disease segmentation (*n* = 24) as 4 or higher on the Likert scale, and 84% (16/19) as 4 or higher with ablation segmentation. It is hoped that this model can provide clinical benefits in the detection of CRLM, the assessment of ablation therapy, and automated planning for radiation therapy.

Our proposed Hybrid-WNet provided fast (<30 s) and accurate segmentation of colorectal liver metastasis and ablation zones, with largely positive physician reviews.

## Data availability statement

The original contributions presented in the study are included in the article/**Supplementary Material**. Further inquiries can be directed to the corresponding author.

## Ethics statement

The studies involving human participants were reviewed and approved by the Institutional Review Board of the University of Texas MD Anderson Cancer Center and registered under the clinical trial #NCT04083378. The patients/participants provided their written informed consent to participate in this study.

## Author contributions

Authors provided valuable insight into the development of the project. Oversight and input in the results, and contributions to the creation of the manuscript. All authors contributed to the article and approved the submitted version.

## Funding

Funding from the Society of Interventional Radiology Foundation Allied Scientist Grant, NIH (NCI R01CA221971, NCI R01CA235564), and the Helen Black Image Guided Fund.

## Acknowledgments

The authors would like to acknowledge the Editing Services, Research Medical Library at The University of Texas MD Anderson Cancer Center.

## Conflict of interest

The authors declare that the research was conducted in the absence of any commercial or financial relationships that could be construed as a potential conflict of interest.

## Publisher’s note

All claims expressed in this article are solely those of the authors and do not necessarily represent those of their affiliated organizations, or those of the publisher, the editors and the reviewers. Any product that may be evaluated in this article, or claim that may be made by its manufacturer, is not guaranteed or endorsed by the publisher.

## Abbreviations

CRC, colorectal cancer; CRLM, colorectal liver metastasis; LRT, loco-regional therapies; CECT, contrast enhanced computed tomography; LiTS, liver tumor segmentation; DSC, dice similarity coefficient, computed tomography; ML, machine learning.
