# Supplementary material for: Automated segmentation of colorectal liver metastasis and liver ablation on contrast-enhanced CT images
Source: Front Oncol. 2022 Aug 11;12:886517. doi: 10.3389/fonc.2022.886517 (PMC9403767; doi:10.3389/fonc.2022.886517)
Supplement: Supplementary file 1 [file Image_1.pdf]

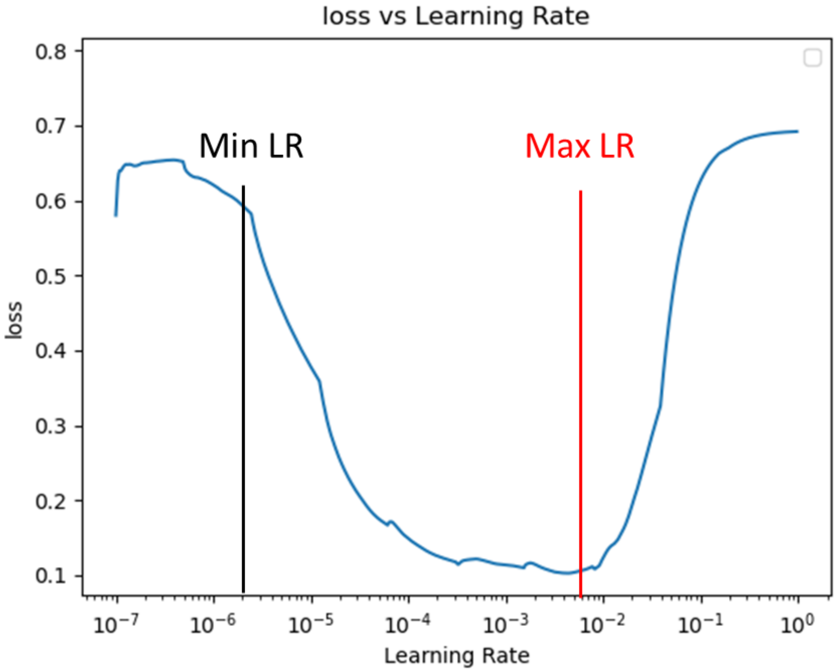

Supplementary Figure 1: Learning rate finder figure. The learning rate is gradually increased from 10e-7 to 10e-0; the point where loss begins to decrease is the minimum learning rate (min LR), and the point where it begins to increase is the maximum learning rate (max LR).
