# Supplementary material for: Automated segmentation of colorectal liver metastasis and liver ablation on contrast-enhanced CT images
Source: Front Oncol. 2022 Aug 11;12:886517. doi: 10.3389/fonc.2022.886517 (PMC9403767; doi:10.3389/fonc.2022.886517)
Supplement: Supplementary file 2 [file Image_2.pdf]

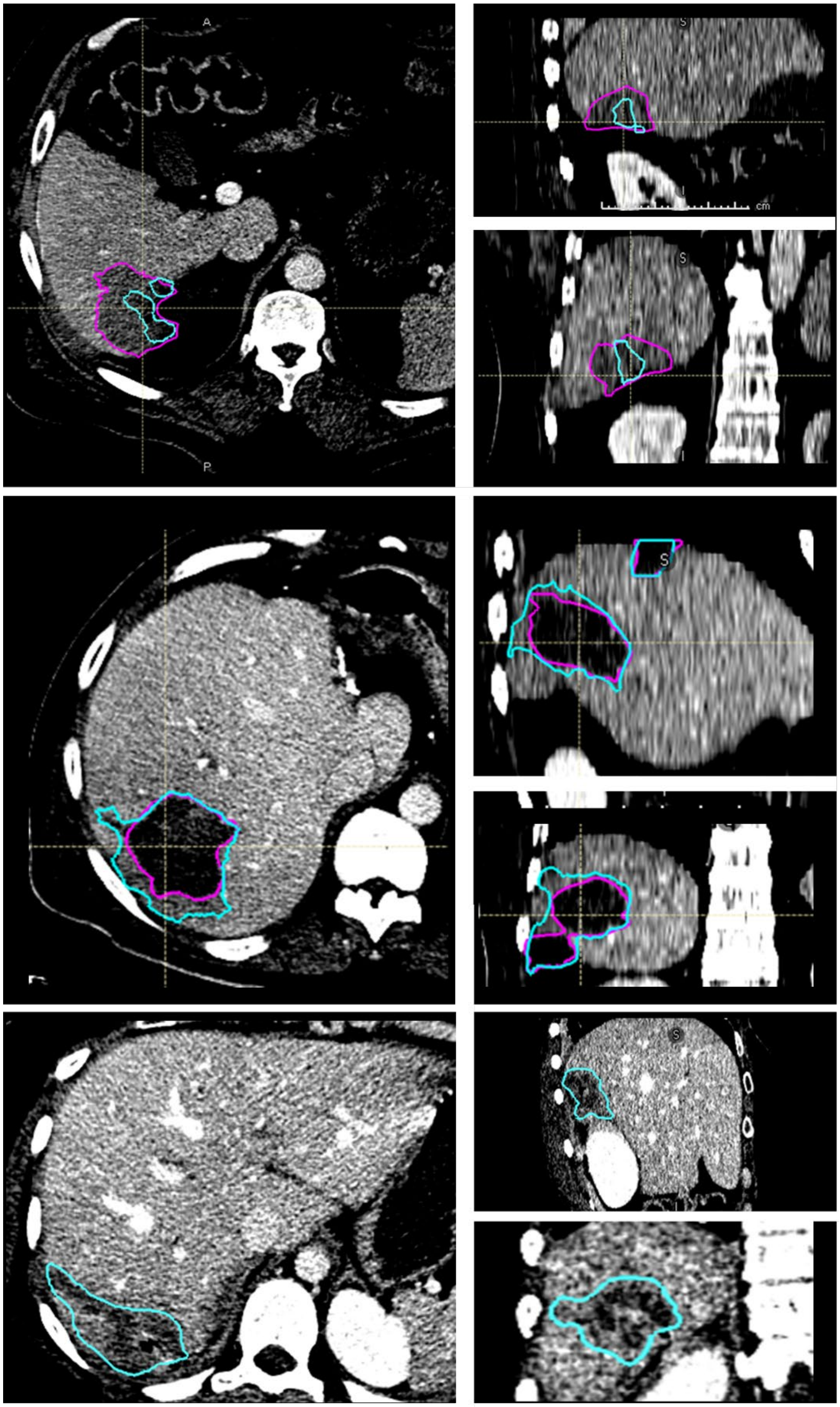

Supplementary Figure 2: Three cases where majority voting had a Likert Score less than 4. (Top) We believe that the top failed due to similarity in appearance to the gallbladder. (Middle, Bottom) Model over-segmented regions around the ablation zones.
