# Supplementary material for: Automated segmentation of colorectal liver metastasis and liver ablation on contrast-enhanced CT images
Source: Front Oncol. 2022 Aug 11;12:886517. doi: 10.3389/fonc.2022.886517 (PMC9403767; doi:10.3389/fonc.2022.886517)
Supplement: Supplementary file 3 [file Table_1.docx]

**Supplementary Table 1: Likert Scoring for Assessment of Colorectal Liver Metastasis (CRLM) and Ablation Contours**

| **Score** | | **Criteria** |
| --- | --- | --- |
| 5 | Excellent | Negligible changes, could be used clinically without edits |
| 4 | Good | Minor changes on < 4 slices or changes that would take < 10 seconds to fix |
| 3 | Fair | Edits needed on multiple slices, but < 50% |
| 2 | Poor | Edits needed on > 50% of slices |
| 1 | Very poor | Contours are poorly predicted, missed disease |
