# Supplementary material for: Automated segmentation of colorectal liver metastasis and liver ablation on contrast-enhanced CT images
Source: Front Oncol. 2022 Aug 11;12:886517. doi: 10.3389/fonc.2022.886517 (PMC9403767; doi:10.3389/fonc.2022.886517)
Supplement: Supplementary file 4 [file Table_2.docx]

**Supplementary Table 2: Breakdown of Likert Score (1-5) for Predicted Contours of CLM and Ablation Sites by Each Reviewer**

|  | **CRLM Scoring (n = 24)** | | | |
| --- | --- | --- | --- | --- |
|  | **Reviewer 1** | **Reviewer 2** | **Reviewer 3** | **Majority Vote** |
| Mean | 4.9 | 4.7 | 4.6 | 4.8 |
| Min | 4 | 4 | 4 | 4 |
| Max | 5 | 5 | 5 | 5 |
| % ≥ 4 | 100 | 100 | 100 | 100 |
|  | **Ablation Scoring (n = 19)** | | | |
|  | **Reviewer 1** | **Reviewer 2** | **Reviewer 3** | **Majority Vote** |
| Mean | 4.5 | 4.1 | 3.8 | 4.1 |
| Min | 3 | 2 | 2 | 2 |
| Max | 5 | 5 | 5 | 5 |
| % ≥ 4 | 95 | 89 | 74 | 84 |

% ≥ 4 is the percentage of cases with a Likert score of ≥ 4.
